# RESIC: A Tool for Comprehensive Adenosine to Inosine RNA Editing Site Identification and Classification

**DOI:** 10.3389/fgene.2021.686851

**Published:** 2021-07-23

**Authors:** Dean Light, Roni Haas, Mahmoud Yazbak, Tal Elfand, Tal Blau, Ayelet T. Lamm

**Affiliations:** Faculty of Biology, Technion – Israel Institute of Technology, Haifa, Israel

**Keywords:** SARS-CoV-2, ADAR, epitranscriptome, interferon, hyper-editing

## Abstract

Adenosine to inosine (A-to-I) RNA editing, the most prevalent type of RNA editing in metazoans, is carried out by adenosine deaminases (ADARs) in double-stranded RNA regions. Several computational approaches have been recently developed to identify A-to-I RNA editing sites from sequencing data, each addressing a particular issue. Here, we present RNA Editing Sites Identification and Classification (RESIC), an efficient pipeline that combines several approaches for the detection and classification of RNA editing sites. The pipeline can be used for all organisms and can use any number of RNA-sequencing datasets as input. RESIC provides (1) the detection of editing sites in both repetitive and non-repetitive genomic regions; (2) the identification of hyper-edited regions; and (3) optional exclusion of polymorphism sites to increase reliability, based on DNA, and ADAR-mutant RNA sequencing datasets, or SNP databases. We demonstrate the utility of RESIC by applying it to human, successfully overlapping and extending the list of known putative editing sites. We further tested changes in the patterns of A-to-I RNA editing, and RNA abundance of ADAR enzymes, following SARS-CoV-2 infection in human cell lines. Our results suggest that upon SARS-CoV-2 infection, compared to mock, the number of hyper editing sites is increased, and in agreement, the activity of ADAR1, which catalyzes hyper-editing, is enhanced. These results imply the involvement of A-to-I RNA editing in conceiving the unpredicted phenotype of COVID-19 disease. RESIC code is open-source and is easily extendable.

## Introduction

The conversion of adenosine to inosine (A-to-I) in double-stranded RNA regions, by adenosine deaminases (ADARs) enzymes, is the most common form of RNA editing in metazoans (Bazak et al., 2014). This type of RNA editing is crucial for normal development of an organism and has a major role in the innate immune response (Mannion et al., 2014; Ganem and Lamm, 2017; Eisenberg and Levanon, 2018). It was shown that changes in editing events are correlated with several types of diseases; including cancer (Maas et al., 2006; Galeano et al., 2012; Gallo and Locatelli, 2012; Kung et al., 2018). Editing sites may serve as biomarkers for cancer and ADAR enzymes are considered as promising gene therapy agents to fight cancer (Ganem et al., 2017). In addition, ADARs are known to be involved in regulation of innate immune response by blocking the interferon (IFN) response upon viral infection (Quin et al., 2021). For these reasons, A-to-I RNA editing is an extensively studied research field in many organisms, and identification of editing sites is of major interest.

In recent years, many efforts have been invested in developing computational approaches to detect A-to-I RNA editing sites from sequencing data (Pinto and Levanon, 2019). Since inosine is very similar in structure to guanosine, inosine is interpreted as guanosine by polymerases during sequencing. This enables the detection of editing sites by comparing between DNA and RNA sequences, to track adenosine to guanosine (A-to-G) mismatches. However, the detection should be carefully performed to avoid false reports due to sequencing and alignment mistakes, alterations in sequence originated from polymorphism, somatic mutations, or other changes which are not the result of editing events (Pinto and Levanon, 2019). The problem is exacerbated by the fact that editing in humans frequently occurs in repetitive regions (Athanasiadis et al., 2004; Blow et al., 2004; Kim et al., 2004; Levanon et al., 2004, 2005; Barak et al., 2009; Kleinberger and Eisenberg, 2010; Osenberg et al., 2010; Paz-Yaacov et al., 2010; Wu J. et al., 2011), which tend to cause alignment errors (Treangen and Salzberg, 2011).

Several tools developed to detect A-to-I RNA editing sites are based on comparison between RNA-seq reads and DNA reference genome. Among these tools are REDItools, which suggest simple comparison using samtools (Picardi and Pesole, 2013), and GIREMI that focused on distinguishing between SNPs and editing, relying on existing SNP databases and a given RNA-seq data (Zhang and Xiao, 2015). Some tools support a direct comparison between RNA-seq reads and DNA reads from the same source, allowing editing site identification without the need for previous knowledge (Picardi and Pesole, 2013; Lee et al., 2015; Wang et al., 2016; Piechotta et al., 2017). A major advantage in comparing between DNA and RNA sequences of the same biological sample is the ability to increase accuracy by excluding changes deriving from unpublished SNPs (Pinto and Levanon, 2019). Another way to increase the results accuracy is parallel comparison between several RNA-seq datasets of several individuals, while taking into consideration that true editing sites would appear in all or most samples (Ramaswami et al., 2013; Wang et al., 2016; Goldstein et al., 2017).

Hyper-editing by ADAR enzymes, which is defined as multiple A-to-I editing sites in a proximity, is a widespread phenomenon. Since most tools designed to identify editing sites are based on the detection of a limited number of mismatches in read alignments (to reduce alignment errors and running time), hyper-editing events, which result in multiple mismatches in a single read (SR), are usually unexposed. Therefore, several recent methods were specially oriented to track hyper-editing sites. Wu D. et al. (2011) and Porath et al. (2014) developed methods that are based on the conversion of unmapped read-sequences to a three-base code genome and thus enable identification of hyper-editing sites. Namely, all As are transformed to Gs in the reference genome and in the RNA-seq reads that previously failed to align, and realignment is then carried out. Following reversion to original sequences, hyper-editing sites, which are rich with A-to-G mismatches, can be located. In both studies, conversion to a three-base code was repeated for all possible nucleotide pairs. It was shown that A-to-G editing was enriched over the other editing types.

Despite the efforts to develop computational tools for A-to-I RNA editing site detection from sequencing data, to date there is not a single platform enabling robust detection of editing sites of different classes and their classification. Here, we present RNA Editing Sites Identification and Classification (RESIC), which enables detection and classification of A-to-I RNA editing sites of different types in a single tool. We expanded the pipeline we previously applied to identify editing sites in repetitive and non-repetitive regions (Goldstein et al., 2017) and adopted the method by Wu D. et al. (2011) and Porath et al. (2014) to find hyper-editing sites. The tool includes an alignment-graph of distinctive architecture and several filtration steps to reduce false identifications. RESIC also enables distinguishing between polymorphism and editing events to increase reliability, by using DNA sequences, ADAR mutant RNA-sequencing datasets, or a SNP database. We demonstrate the utility of RESIC by applying it to mapping A-to-I RNA editing sites in 16 human tissues, from the Illumina Human Body Map project, analyzed for a similar purpose by others (Zhu et al., 2013; Bazak et al., 2014; Porath et al., 2014). Our analysis reproduced known putative editing sites, detected by others and included in the RADAR database (Ramaswami and Li, 2014), and extended the list of known sites.

Since aberrant IFN and cytokine responses were observed in COVID-19 patients (Moore and June, 2020) and ADAR1 was shown to activate the IFN reaction (Baños-Lara et al., 2013), we further interrogate the activity of A-to-I RNA editing upon SARS-CoV-2 infection. We show that in SARS-CoV-2 infected samples, compared to mock, ADAR1 is the only A-to-I RNA editing enzyme that is differentially expressed, and the numbers of A-to-I hyper editing sites are larger.

## Methods and Definitions

### RNA Editing Sites Identification and Classification Algorithmic Definitions

RNA Editing Sites Identification and Classification enables the user to supply DNA or RNA datasets that should exhibit the desired editing phenomena and DNA or RNA sequencing datasets that should not exhibit the desired editing phenomena. The latter group is used to exclude changes deriving from SNPs. Since nucleotide changes in the former sequencing datasets correspond to positive evidence of that sites undergoing editing and the latter datasets correspond to negative evidence, we term these sets of datasets as positive and negative datasets. RESIC is completely reference agnostic. The users provide whichever reference file they wish to use for the alignment as well.

### Ambiguous Read Filtering

For ambiguous read filtering, we adopted the method of Porath et al. (2014). Briefly, we filtered out the reads that meet the next criteria: one or more nucleotides represent over 60% or under 10% of the read sequence, more than 10% Ns (when a base call could not be done), average Phred quality score < 25, and more than 20 repeats of a single nucleotide in a row.

### Alignment Scheme

We define an alignment scheme to be a 4-tuple *S* = (*A*,*p*,*f*_1_,*f*_2_) where *A* is an alignment algorithm, *p* is a list of alignment parameters for *A*, *f_1* is a preprocessing function of the raw datasets and *f_2* is a postprocessing function for aligned and misaligned reads. These seemingly verbose definitions enable RESIC to decouple the choice of alignment algorithm from the rest of the modules in RESIC.

Let *S* be an alignment scheme, *L* be a sequencing dataset and *R* be a genome reference, we define *P*_*S,L,R*_ and *N*_*S,L,R*_to be the aligned and misaligned read fractions resulting from running S on L and R. We define S(*L*,*R*) = (*P*_*S*,*L*,*R*_,*N*_*S*,*L*,*R*_).

We say that a scheme is normal if *f_1* and *f_2* are identity functions in said scheme. Pseudo code for calculating S(L,R):

def S(L,R):

L′,R′=f_1(L,R) # preprocessing the

sequencing

datasets

P′,N′=A(L′,R′,p) # Alighning the sequencing

datasets

P,N = f_2(P′,N′) # post processing results

return P,N

### Graph Aligner

Given a directed acyclic graph *G* = (*V*,*E*) where nodes in *V* are alignment schemes, *L* a sequencing dataset and *R* a reference we define new alignment schemes *G*_*v*_(*L*,*R*) for each *v* ∈ *V* to be defined as follows:

$$G_{v}\,\left(L,R\right)=\left\{ \begin{array}{c} \begin{array}{cc} v\,\left(L,R\right) & I\,n\,\left(v\right)=\emptyset \end{array}\\ \begin{array}{cc} v\,\left(L^{\prime \prime },R\right) & L^{{}^{\prime \prime }}=\cap_{u\vee \left(u,v\right)\in E}N_{G_{u},L,R} \end{array} \end{array}\right.$$

We define *G*(*L*,*R*) to be a set of aligned sequencing datasets {*P*_*G*_*v*_,*L*,*R*_|*v* ∈ *V*}. Simply put, we perform the alignment scheme of node v on all read fragments that were misaligned in any of v’s ancestors.

### 3nt Genome Alignment Scheme

Let *X* and *Y* be two distinct nucleotides. To be able mapping hyper editing sites, we apply the 3nt alignment scheme by which each appearance of either *X* or *Y* is transformed into *X* in both the sequencing datasets (reads) and the reference genomes. That was similarly done by others (Wu D. et al., 2011; Porath et al., 2014). However, we present an advanced 3nt technique to map hyper antisense reads as was not described elsewhere, to the best of our knowledge. First, for each *X* and *Y* nucleotides pairs, we first apply the scheme to the reads at the given node (see section “Graph Aligner”) and to the reference sense strand. Next, in order to identify hyper editing sites on the antisense strand, for each *X* and *Y* nucleotides pairs, we create the complement reference genome, based on the original reference, and reverse the reads that were unmapped in the previous step, to achieve the 3′–5′ direction, same as the created reference. Then we reapply the 3nt alignment scheme while considering the manipulation of the reference genome and reads when recording the mapped reads as aligned to the antisense.

In each step, after mapping the reads, aligned and unaligned reads are reverted to their original sequence *via* custom python scripts. Supplementary Figure 1 illustrates in details the 3nt genome alignment scheme. To conduct the 3nt scheme we use awk (Aho et al., 1996) and sed (Free Software Foundation, 2019).

### Site Filtering

After performing the graph alignment for each of the given sequencing datasets, samtools (Li et al., 2009) is used to convert the files into pileup format. Then, several filtering steps are performed as detailed below. All parameters (*l*, *k1*, *k2*, *u*, *r*, and *c*) are user defined.

First, sites with no nucleotide changes and sites covered by less than *l* reads are discarded. We discarded sites from the positive datasets if those same sites appeared in any negative dataset with a nucleotide change.

### Editing Percent Filtering

For each positive sequencing dataset, we filter out any site: (1) whose most abundant nucleotide change constitutes less than *k_1* percent or more than *k_2* percent of the reads mapped to that site, (2) whose other nucleotide changes constitute over *u* percent of the reads mapped to that site, and (3) whose most abundant nucleotide change is in at least *r* reads. We term: *k_1*, the minimal editing percent threshold, *k_2*, the maximal editing percent threshold, *r*, the editing read threshold, and *u*, the editing noise threshold.

### Unique Site Filtering

We filter all sites that were defined as editing sites at the previous step, under more than one editing category (e.g., non-repetitive and hyper non-repetitive A to C), if they represented more than one type of nucleotide change (e.g., once A to G and the other time A to C).

### Hyper Editing Filtering

Deriving from our method, it may be possible that under the hyper editing categories, a non-hyper editing site would be recorded. Namely, for each pair of nucleotides X and Y that we perform the 3nt genome scheme, other nucleotide mismatches than hyper X to Y or Y to X may be noted, enabled by the new conditions created by the 3nt scheme. Therefore, we filter the hyper editing files to include only X to Y or Y to X changes (see an illustration in Supplementary Figure 1).

## Consensus

We filter out any sites that are not present in over *c* percent of positive datasets.

### A-to-I Editing Identification Pipe

We implemented a hyper editing alignment scheme and built an alignment graph that could target any editing type. Specifically, in the analysis described here we only applied RESIC to A-to-I RNA editing.

### A-to-I RNA Editing Alignment Graph

In our screen for A-to-I editing sites, we define two classes of alignment schemes, non-repetitive alignment for reads that map uniquely to the genome and repetitive for repetitive regions or regions that cannot be differentiated by our reads *via* alignment. Our graph alignment, summarized in Supplementary Figure 2, is as follows: we align sequencing datasets using the non-repetitive normal scheme followed by the repetitive normal scheme. Then we branch out and for each pair of distinct nucleotides X and Y, we perform the non-repetitive 3nt genome scheme, and the repetitive 3nt genome scheme.

### RNA Editing Profiling of Illumina BodyMap2 Transcriptome

RNA-seq datasets from 16 human tissues (Illumina Human Body Map 2.0 Project; GEO accession number GSE30611) that were sequenced at 75 SR, were downloaded from SRA. FastQC was used to control the read quality and trimming was performed accordingly. Reads were further collapsed and then taken for a RESIC run. For the underlying sequencing algorithm, we used Bowtie (Langmead et al., 2009) alignment tool. For the non-repetitive and repetitive alignments, we configured bowtie to align to fragments if they map to under 2, or 20 different genomic locations, respectively, with at most 3 single base mismatches and to consider matches for a read r as the set of alignment results for r with the smallest alignment score (-m 2 -n 3 –best –strata, - l 50 –chunkmbs 200, and -m 20 -n 3 –best –strata - l 50 –chunkmbs 200, respectively). Similar alignment was used in Goldstein et al. (2017). For the site filtration steps, we choose *l* = 2 to be the coverage per site threshold, *k*_1_=30 and *k*_2_=99 for the editing minimal and maximal percent threshold, respectively, *u* = 3 for the noise thresholds, *r* = 2 for the editing read threshold, and *c* = 0 for consensus threshold. The site lists obtained for each tissue were filtered to have only A-to-I sites, namely A-to-G, or T-to-C mismatches in both strands.

The list of obtained editing sites was compared to the entire list from RADAR database (Ramaswami and Li, 2014), We considered as shared editing sites, sites that are included in the RADAR list or sites that have gene annotations similar to the ones appeared in the RADAR list.

### Experimental Validation of Novel Editing Sites

We investigated three novel candidate editing sites that were found in brain tissue using RESIC. For validation using Sanger sequencing, RNA from three sections of brain glioblastoma sample (a kind gift from Dedi Meiri and Yaniv Lerenthal) was used. RNA was treated with turbo DNase I (Invitrogene) and then a reverse transcriptase reaction was performed with Maxima First Strand cDNA Synthesis Kit (Thermo Scientific), using primers that surrounded the candidate editing sites (listed in the Supplementary material). The amplification products were directly sequenced by Sanger sequencing.

### Profiling of SARS-CoV-2 Infected Calu-3 Cells

Raw RNA-seq data of Calu-3 human Lung adenocarcinoma cells infected with SARS-CoV-2 virus or mock, were downloaded from SRA, BioProject PRJNA615032. FastQC was used to control the read quality and trimming was performed accordingly. Reads were collapsed and first aligned to the SARS-CoV-2 reference genome version NC_045512.2 using bowtie. Alignment to the SARS-CoV-2 genome was made to exclude reads that are originated from the virus for further analysis, and to validate that in contrast to the mock samples, the SARS-CoV-2 samples are infected with the virus. Indeed, few thousands of reads were mapped to the SARS-CoV-2 genome, only for the SARS-CoV-2 infected samples. We applied RESIC separately on the unaligned reads of the mock and SARS-CoV-2 infected samples (three biological replicates each) to identify changes in RNA editing events upon coronavirus infection. For the underline sequencing algorithm, we used Bowtie (Langmead et al., 2009) alignment tool. For the non-repetitive and repetitive alignments, we configured bowtie to align to fragments if they map to under 2, or 100 different genomic locations, respectively, with at most three single base mismatches (-m 2 -n 3 –best –strata, - l 50 –chunkmbs 200, and -m 100 -n 3 –best –strata - l 50–chunkmbs 200, respectively). For the site filtration steps, we choose *l* = 2 to be the coverage per site threshold, *k*_1_=30 and *k*_2_=99 for the editing minimal and maximal percent threshold, respectively, *u* = 3 for the noise thresholds, and *r* = 2 for the editing read threshold. The consensus module was run with *c* = 0.5. We then filtered the site lists obtained to have only A-to-I sites. Since the RNA library preparation strategy was stranded (the sequenced strand must be from the actual expressed strand), we filtered the files obtained to include only actual A-to-G sites and not T-to-C. To test the difference in the numbers of editing sites, under the non-repetitive and hyper-non-repetitive classes, between all SARS-CoV-2 and mock samples, we performed Two-tailed *T*-test with equal variances (to determine equal variances Levene Test was performed). For this test, we included the normalized (according to the total read coverage per each class stage) numbers of editing sites of six repeats for each sample type, originated from 3 biological replicas that were evaluated twice for each strand separately.

To perform differential expression analysis (DEA), we mapped the same unaligned reads that were used for RESIC analysis before, to the human transcriptome version GRCh37 (hg19) using bowtie. Gene expression levels were evaluated by read counts. We then compared our created gene counts to the already processed counts downloaded from GEO: GSE147507. Although read count values were not identical, as expected due to the use of different alignment tools, the trend was the same.

We performed DEA, using DESeq2 (Love et al., 2014), with lfcShrink function and apeglm shrinkage estimator type.

### RNA Editing Sites Identification and Classification Code Availability

RNA Editing Sites Identification and Classification is an open source available at our GitHub repository^1^.

## Results and Discussion

### RNA Editing Sites Identification and Classification–A Comprehensive Tool for Identification RNA Editing Sites

**FIGURE 1:**
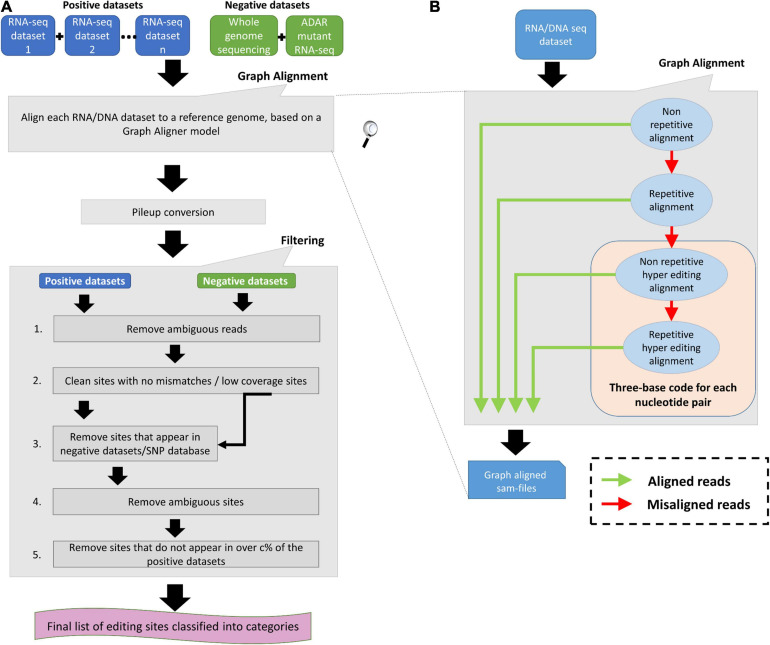
RNA Editing Sites Identification and Classification (RESIC’s) schematic view. **(A)** Overall description of the RESIC pipeline. First all given sequencing datasets are filtered for ambiguous reads and go through the graph alignment scheme to detect A-to-I editing sites of different classes. The RNA-seq datasets intended for editing-sites identification are termed positive datasets, and the RNA and/or DNA sequencing datasets used to contradict editing-site existence are termed negative datasets. Sam-files for each alignment node are created using a sequence aligner (Langmead et al., 2009) and converted into pileup files using samtools (Li et al., 2009). The next stage includes several filtering steps for removing: (1) sites with no changes compared to the reference or low coverage sites; (2) SNPs or mismatches that are not originated in RNA editing, based on comparison to the negative datasets or/and a SNP database (optional); (3) sites with more than one prominent mismatch (large noise) or with low change ratio; and (4) sites that do not appear in over c% samples (optional). Finally, a list of editing sites divided into classes are given as an output with descriptive plots. **(B)** Zoom in on the data flow illustration of the graph aligner model for the four-layer graph used in the study.

**FIGURE 2:**
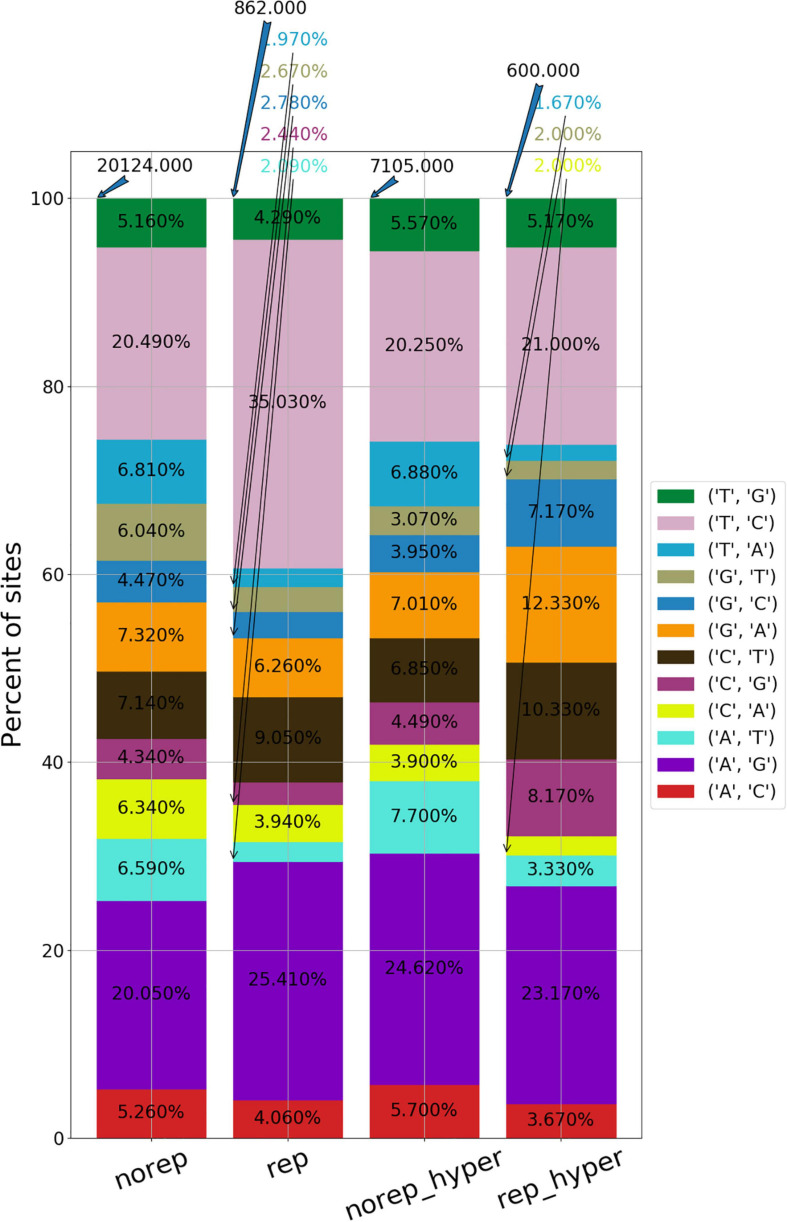
An example for RESIC editing percent distribution plot, obtained for an adrenal tissue sample. Blue arrow at the top of each bar shows the total number of sites being identified for the class. The percentages on the bars present the total number of editing type out of all identified site in the class. The x-axis labels represent the RNA editing classes. Norep: non-repetitive, rep: repetitive, norep_hyper: non-repetitive hyper, rep_hyper: repetitive hyper.

To have a complete identification of RNA editing sites, which include sites in non-repetitive regions, sites in repetitive regions, and sites in hyper-editing regions, we generated a novel tool termed “RESIC.” RESIC composed of an enhanced alignment graph model to identify and classify editing sites by their type, multiple-step filtering process to increase result reliability in a flexible manner (Figures 1A,B) and plots for data visualization (see an example in Figure 2).

To use RESIC, the user should supply positive datasets, i.e., RNA-/DNA sequencing datasets that should exhibit the desired editing phenomena, and a reference genome. The user may also supply negative datasets, DNA or RNA sequencing datasets that should not exhibit the desired editing phenomena, or a SNP database, to contradict editing-site existence.

All datasets are first processed according to the graph alignment (Figures 1A,B). The graph alignment was designed to track editing sites of different types by aligning a given set of reads to the reference genome in a specific parameter configuration setup that represents each editing class. Namely, we demanded unique or multiple alignment to detect non-repetitive and repetitive sites, respectively, Next for sequences that did not align, we converted read-sequences to a three-base code to detect hyper editing sites for all possible nucleotide pairs, as described by Wu D. et al. (2011) and Porath et al. (2014). Our alignment-graph distinctive architecture enables the fluent utilization of an unmapped read-set that was discarded in one alignment level for defining editing sites of a different class in the next level (Figure 1B). This enables the identification of multiple editing-site classes in a single platform. While RESIC was built to provide a way to consolidate the many ongoing efforts at A-to-I editing site identification, our graph aligner model is general and robust enough to stand on its own and contribute to general identification of nucleotide changes. RESIC was based on algorithms and scripts whose ability to correctly identify editing sites was tested experimentally in Wu D. et al. (2011) and Goldstein et al. (2017). Further validation is also presented below.

Following alignment, the candidate editing sites that were identified are going through strict multi-stage filtering process (Figure 1A). The filtering process’ aim is to increase the results’ reliability considering different types of possible errors. In one type, sites in which there is more than one mismatch type or sites showing low change ratio are suspicious as technical errors likely to be formed during sequencing or alignment and discarded due to low reliability. The user may easily modify the limiting thresholds controlling these filtering steps (i.e., minimal coverage per site, minimal change ratio, and maximal noise ratio). For example, filtering out ambiguous reads to reduce alignment errors as well as filtering low covered and noisy (with more than one mismatch) sites. Also, reducing the maximal editing percent threshold to less than 100% can reduce SNPs. In another type, incorrect recognition of SNPs as editing sites can be prevented by excluding sites that show the same nucleotide alterations in both the DNA and the RNA sequences. The user may choose (but it is not mandatory) to supplying DNA sequencing data of the same individual used to detect editing sites, for enabling the described DNA based exclusion. Another way in which SNPs can be distinguished from editing sites is parallel comparison between several samples of different individuals, by testing the consensus level of editing sites. The rationale behind parallel comparison of various individuals is that true editing sites would appear in all or most samples (Ramaswami et al., 2013; Wang et al., 2016; Goldstein et al., 2017). In addition, biological replicas can eliminate changes that occurred because of sequencing errors. Testing for consensus in editing sites among several samples is a less favorable option to eliminate SNPs, in case DNA sequencing dataset is supplied. The user may choose to neutralize the consensus filtering step or modify the consensus threshold.

Our motivation was to build one tool that envelopes several algorithms and enables prediction of all classes of editing sites with as much flexibility as possible. Most of the current bioinformatics tools as described in the introduction focus on one class of editing sites identification (for example, only on hyper editing sites as in Wu D. et al. (2011), with very limited flexibility on the input data, and on the stringency of the detection.

### RNA Editing Sites Identification and Classification Enhanced the Number of Identified A-to-I Editing Sites in Human Tissues

In order to test the utility of the tool, we used RNA-seq datasets from seven human tissues: adipose, adrenal, brain, breast, colon, kidney, and heart (Illumina Human Body Map 2.0 Project; GEO accession number GSE30611) that were sequenced at 75 SR. These datasets were previously screened for editing sites by others (Zhu et al., 2013; Bazak et al., 2014; Porath et al., 2014).

We used the latest GRCh37 SNP database (NCBI) to eliminate changes that are not originated from A-to-I RNA editing, but from genomic polymorphism. All datasets were processed according to the graph alignment and went through all filtration steps (for parameters setup, see “Methods and Definitions”). Since each of the 16 samples is originated from a different tissue, and editing sites may be tissue specific (Picardi et al., 2015), we defined *c* = 0 for consensus threshold. To test the power of RESIC to specifically identify A-to-I editing sites, we compared the output of RESIC (Supplementary Table 1) to the collection of A-to-I RNA editing sites, taken from RADAR (Ramaswami and Li, 2014). It is indicated by our comparison (Table 1) that over 75% of the non-repetitive sites RESIC identified, and over 65% of non-repetitive hyper sites are also included in the RADAR collection. This large overlap is expected, since RADAR is based, among others, on the same samples analyzed by us, and at the same time strengthening the reliability of RESIC. Since hyper editing sites are less frequently found by traditional tools, dictated by the more common alignment parameter setup (Pinto and Levanon, 2019) and tools that aimed for tracking hyper-editing sites (Wu D. et al., 2011; Porath et al., 2014) are less abundant, it is not surprising that a smaller overlap was obtained for non-repetitive hyper sites, compared to non-repetitive.

**TABLE 1:** Overlap levels of the detected A-to-I editing sites with RADAR database.

**RESIC editing class**	**Non-repetitive**	**Non-repetitive hyper**	**Repetitive**	**Repetitive hyper**
Number of A-to-I editing sites by RESIC	23,194	12,496	1,225	895
Shared with RADAR database full list (%)^1^	75.4	65.4	27.0	30.7
Novel sites, non-shared with RADAR database full list (%)	24.6	34.6	73.0	69.3

*^1^We considered sites as “shared with RADAR” if they were either included in the collection taken from RADAR, or their annotated genes are in RADAR’s collection.*

Among all classes defined *via* RESIC, the “non-repetitive” class yielded the largest overlap (75.4%, 65.4%, 27.0%, and 30.7%, for non-repetitive, non-repetitive hyper, repetitive, and repetitive hyper, respectively; Table 1). For repetitive site classes, smaller overlap was obtained.

A substantial portion of sites detected by RESIC were not identified by others. The explanation for the new identified sites in this study may be the result of the usage of different tools for alignment [i.e., Bowtie in our case and BWA, or a combination of Bowtie, SOAP, and BWA in Zhu et al. (2013) and Porath et al. (2014)], as well as various threshold parameters and filtering criteria being set to consider sites as “editing sites” across tools.

The distribution of the editing events divided into classes can be shown in Figure 2, presenting for example the RESIC results for an adrenal tissue sample (the plots obtained from the rest of the samples can be found in the Supplementary Figures 3–8. A-to-G and T-to-C are both considered as editing changes because the data is not stranded. Over all classes being identified according to the graph alignment, A-to-G and T-to-C types were highly enriched, as expected (Figure 2). While non A-to-G mismatches are expected to be uncommon (Li et al., 2011; Kleinman and Majewski, 2012), RESIC still identified a certain amount of sites of that type, although in a much lower extent. This may be the result of rare SNPs that are uncovered by the SNP database being used.

Overall, the unique characterization of RESIC enables the detection of different classes of editing events, in one tool. The specificity of RESIC can be seamlessly controlled by modifying the running parameters, and by suppling datasets to exclude SNPs.

Finally, we validated experimentally using sangar sequencing a few of the most likely novel candidate editing sites that were found using RESIC in the brain tissue. These sites, which were not included in the RADAR collection, are in: chromosome 2, position 130737822, *RAB6C* gene; chromosome 14, position 28733993; chromosome 15, position 39889079, *THBS1* gene. For validation, we used RNA from three sections of brain glioblastoma tissue. For two of the novel editing sites, in chromosome 2 and chromosome 14, a high editing ratio was clearly observed in sanger sequencing, for all tissue sections (Supplementary Figure 9). For the third site on chromosome 15, no editing was observed. However, since not the same cells were used for the bioinformatics analysis and the experimental validation, this test does not disapprove of the existence of a real editing site at that location. In addition, this sample was taken from cancer cells, which were already shown to have differences in editing levels (Ganem et al., 2017). Considering the biological differences between the samples used for the bioinformatics analysis and the experimental validation, the overall support of the validation results in RESIC tool reliability is strong.

### SARS CoV-2 Infection Results in an Extensive A-to-I Hyper RNA Editing and Upregulation of ADAR1 Enzyme

Systemic inflammatory responses to viral infection are triggered by IFN-mediated innate immune response (Schneider et al., 2014). Properly orchestrated, this type of immune response leads to inhibition of virus replication, promotion of virus clearance and induction of tissue repair. However, in some people infected with COVID-19, unpredictably, the innate immune response is exaggerated leading to Acute Respiratory Distress Syndrome (ARDS) (Moore and June, 2020). The innate immune response is regulated by ADAR enzymes, which modulate the IFN response to viral infection and reduce the innate immune response. ADAR1 was also shown to prevent Melanoma Differentiation-Associated Protein 5 (MDA5) from sensing dsRNA (Liddicoat et al., 2015) and activating both type I and type III IFNs (Baños-Lara et al., 2013). Therefore, we wished to interrogate the activity of ADAR enzymes following SARS-CoV-2 infection. For this purpose, we analyzed the data of Blanco-Melo et al. (2020), of human Calu3 cells infected with SARS-CoV-2 virus or mock, to examine the differences in A-to-I RNA editing patterns.

**FIGURE 3:**
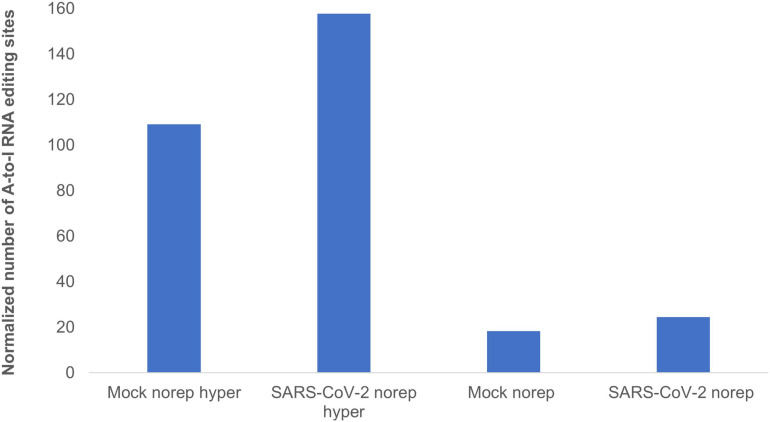
A-to-I hyper editing sites are more frequent in SARS-CoV-2 infected cells compared to mock. Presented here are the numbers of A-to-I RNA editing sites detected in SARS-CoV-2 or Mock samples in total, normalized to total counts. Norep: non-repetitive editing sites, norep hyper: hyper non-repetitive editing sites. The number of non-repetitive hyper editing sites is significantly higher in SARS-CoV-2 samples compared to mock (*P*-value = 0.0371), and the number of non-repetitive sites is similar in both SARS-CoV-2 and mock (*P*-value > 0.05).

To identify and classify RNA editing sites, we applied RESIC on Calu3 cell lines that were infected with SARS-CoV-2 virus or mock (see “Methods and Definitions”). Following RESIC run, we assessed the numbers of the most prevalent classes of A-to-I RNA editing types: non-repetitive, and hyper non-repetitive. We compared between SARS-CoV-2 and mock editing site numbers for each class following normalization, relying on the total read processed in each node. A-to-I non-repetitive hyper editing was significantly more frequent in SARS-CoV-2 infected cells compared to mock (*P*-value = 0.0371). Overall, the number of hyper editing sites upon SARS-CoV-2 infection was 36.45% greater than in mock (Figure 3). For the non-repetitive class, the site numbers were relatively low for both sample types. The number of non-repetitive editing sites was larger as well in SARS-CoV-2, but the difference was not significant (*P*-value = 0.0765). Overall, the number of non-repetitive sites upon SARS-CoV-2 infection was 28.62% greater than in mock (Figure 3).

We next wished to further validate the editing sites that we found in human adenocarcinomic lung epithelial (Calu3) cells that were infected with SARS-CoV-2, using a different human cell line. For that aim, we used RNA-seq data from human adenocarcinomic alveolar basal epithelial (A549) that were infected with SARS-CoV-2 virus (Blanco-Melo et al., 2020) and searched for RESIC predicted sites in these cells. Only two biological replicas from A549 samples had enough sequencing coverage to search for editing sites. Using these two samples, we were able to detect (in A549 cell line) 52% of the non-repetitive sites, and 37% of the hyper-non-repetitive sites that we found in SARS-CoV-2 calu3 cells. This large overlap of editing sites, which was obtained despite a low amount of RNA-seq reads among the A549 analyzed samples and the comparison between distinct types of cells (which are expected to have different editing sites), is encouraging.

Another goal was to validate the novel editing sites found by us, using the BodyMap2 dataset, that include seven different tissues: adipose, adrenal, brain, breast, colon, kidney, and heart. For that purpose, we searched for the new identified sites (Supplementary Table 1) in mock, A549, and Calu3 cell lines. Out of the full list of non-repetitive new editing sites that we found, 26.9% were also detected under the same category (non-repetitive) in A549 and Calu3 cells, although the cell lines are originated from the lungs that were not represented in our original analysis.

To understand whether the higher editing activity upon SRAS-CoV-2 infection is manifested by a larger number of sites in the same genes as in mock, or additional sites located in new genes, we characterized the editing landscape with respect to site locations and gene annotation.

We first tested the ratio of shared sites between SARS-CoV-2 and mock samples. For both non-repetitive and hyper non-repetitive classes, almost ∼70% of the sites were unique among samples infected with SARS-CoV-2, and about 60% of the sites were unique for mock samples (Supplementary Table 2). We next annotated the unique sites for each sample type, across different classes. It was apparent that the answer for our initial question is that among the unique editing sites following SARS-CoV-2 infection, some of the sites were extended the editing in baseline genes (considering the mock as the baseline), but most of the sites were in new genes (Supplementary Table 2).

Interestingly, the gene APOBEC3C became hyper edited following SARS-CoV-2 infection (Supplementary Table 3), while in mock samples it was classified under the non-repetitive class. The APOBEC family of enzymes edits C-to-U RNA modifications and known to be involved in regulation of innate immune response (Rosenberg et al., 2011; Schaefer et al., 2017). C-to-U editing of antibody-coding genes in the host’s DNA leads to diversification of the repertoire of antibodies produced against viruses, called somatic hypermutation (SHM) (Cogné, 2013). Therefore, hyper editing in APOBC genes may indicate for their involvement in COVID-19 phenotype, as part of a complex immune regulation system, controlling by A-to-I RNA editing.

To test if the sets of unique edited genes display shared biological processes, we examined their biological process enrichment, using the web-based tool GeneMANIA (Zuberi et al., 2013). Submitting the list of hyper-non-repetitive unique edited genes, upon SARS-CoV-2 infection, resulted in the enrichment of processes related to the regulation of I-kappaB kinase/NF-kappaB signaling (*P*-adjusted values of related pathways in the range of 1.91E-03–3.50E-04; Supplementary Table 3). This result is intriguing in the light of strong indications suggesting that NF-kappaB pathway signaling has a critical role in controlling an excessive immune activation and ARDS (Kircheis et al., 2020). These indications, together with our result of hyper-editing in genes participating in the NF-kappaB pathway, suggest that A-to-I RNA editing activity may be critical to define the progression of COVID-19 disease and the risk to develop ARDS. We next, tested an enrichment for genes that were classified under the non-repetitive class and were uniquely edited in SARS-CoV-2 samples. Strong enrichment was obtained for processes related to IFN response (*P*-adjusted values of related pathways in the range of 1.25E-06–1.03E-12; Supplementary Table 4), corroborating previous evidence that ADARs control IFN activation under viral infections (Baños-Lara et al., 2013), and suggests particularly that in COVID-19, ADARs control the level of immune response. We further run GeneMANIA for biological process enrichment with the mock unique gene sets, as a control. No processes related to IFN or NF-kappaB signaling were enriched in FDR < 0.05 (Supplementary Tables 5, 6).

Given these observations, we reasoned that interrogating the differences in ADAR RNA expression levels between SARS-CoV-2 and mock treated samples, would help to complete the picture. Therefore, we performed DEA, on the same data of Blanco-Melo et al. (2020) used for RESIC. We first created the read counts for both SARA-CoV-2 and mock Calu3 samples (see “Methods and Definitions”). We then compared our created gene counts to those reported by Blanco-Melo et al. (2020) GEO accession number GSE147507. Although read count values were not identical, as expected due to the use of different alignment tools, the trend was the same.

**FIGURE 4:**
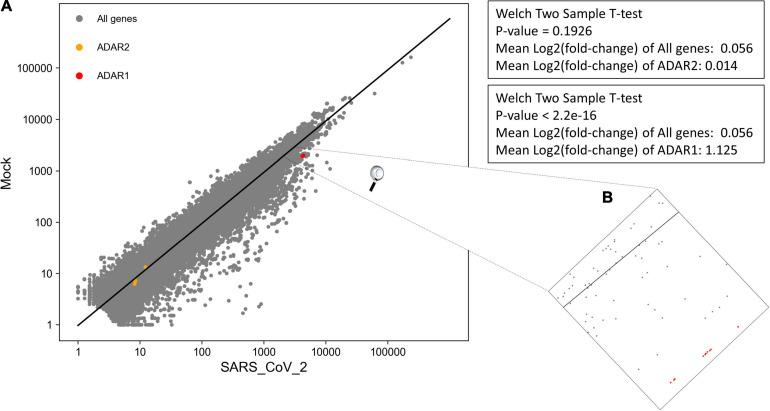
Significant upregulation of ADAR1 isoforms, but not ADAR2, in cells infected with SARS-CoV-2 virus, compared to mock. **(A)** Log scale plot shows normalized gene counts from mock cells against cells infected by SARS-CoV-2 virus. Every dot in the graph represents a gene: ADAR1 isoforms (red), ADAR2 isoforms (orange), and all other genes (gray). The black line is the regression line for all genes. The *P*-values were obtained using a Welch two-sample *T*-test on only transcripts with coefficient variation > 1. **(B)** Zoom in on ADAR1 isoforms on the log scale plot. All 10 isoforms are closely located on the plot.

We performed DEA to identify changes in the expression of ADAR genes and A-to-I RNA editing, following SARS-CoV-2 infection (Supplementary Table 7). We found that all 10 ADAR1 isoforms are significantly upregulated Figure 4, *P*-value = 2.2e-16) in SARS-CoV-2 Calu3 infected cells. In comparison, the expression of ADAR2 did not differ between SARS-CoV-2 and mock samples (Figure 4, *P*-value > > 0.05). We also found that *IFIH1* (NM_022168.4), that encodes for MDA5 is significantly up-regulated in the SARS-CoV-2 infected samples (Supplementary Table 7, *P*-adjusted = 7.46e-137). This is in line with findings indicating that the IFN response upon SARS-CoV-2 infection is primarily regulated by MDA5 (Yin et al., 2021). Since ADAR1 is known to prevent MDA5 from sensing dsRNA (Liddicoat et al., 2015), this result strengthens the conclusion that ADAR1 is largely involved in the immune response following SARS-CoV-2.

Collectively, we suggest that upon SARS-CoV-2 infection, compared to mock (1) the number of hyper editing sites is increased; and (2) ADAR1 activity is enhanced. The combination between these two observations goes together with the finding that ADAR1 is the enzyme mostly catalyzing hyper editing sites (Porath et al., 2014).

We tested if these results hold true for more *in vitro* SARS-CoV-2 infected cell types created in the same study. For that purpose, we downloaded from GEO the already processed gene count data, for A549 and NHBE cells infected with SARS-CoV-2 high-multiplicity of infection (MOI). We chose downloading the already processed gene count data after validating for Calu3 cells that the gene count values created by us and the downloaded gene count from GEO (accession number GSE147507) are of the same trend (see “Methods and Definitions”). For A549 cells, with a vector expressing human ACE2, indeed ADAR1, but not ADAR2, was significantly upregulated following SARS-CoV-2 infection, corroborating our previous results for Calu3 cells. However, for NHBE cells, both ADAR1 and ADAR2 were not significantly changed after SARS-CoV-2 infection (Supplementary Table 8). The non-significant upregulation of ADAR1 in the last case may be because of the different cell types used. In any event, we concluded that unlike ADAR2 the expression of ADAR1 is substantially different upon SARS-CoV-2 infection, at least in some cell types.

Taken together, our results suggest that the catalyzation of hyper editing sites by ADAR1 is enhanced following SARS-CoV-2 infection. These results are intriguing in the context of ADAR1’s role to block the IFN response, and particularly the role of hyper editing events to suppress the IFN induction (Vitali and Scadden, 2010). We hypothesize that editing levels might be indicative of the progression of COVID-19 disease and the risk to develop ARDS, as holds true in autoimmune diseases, due to the editing effect on the IFN response. Therefore, these results shed new light on the involvement of A-to-I RNA editing mechanism in COVID-19 disease. We note that supporting experimental validation is required to assess our conclusions. Our analysis encourages further exhaustive study of A-to-I RNA editing role in COVID-19 disease.

## Data Availability Statement

The original contributions presented in the study are included in the article/Supplementary Material, further inquiries can be directed to the corresponding author.

## Author Contributions

DL, RH, and ATL conceived and designed the study and wrote the manuscript with input from all authors. DL, RH, MY, TE, and TB implemented and developed RESIC. RH analyzed the data. ATL supervised the work. All authors contributed to the article and approved the submitted version.

## Conflict of Interest

The authors declare that the research was conducted in the absence of any commercial or financial relationships that could be construed as a potential conflict of interest.

## Publisher’s Note

All claims expressed in this article are solely those of the authors and do not necessarily represent those of their affiliated organizations, or those of the publisher, the editors and the reviewers. Any product that may be evaluated in this article, or claim that may be made by its manufacturer, is not guaranteed or endorsed by the publisher.

## Footnotes

1 https://github.com/Lammlab/Resic

## References

[B1] Aho A. Kernighan B. Weinberger P. (1996). *Awk A Pattern Scanning and Processing Language*, Second Edn. Murray Hill, NJ: Bell Laboratories.

[B2] Athanasiadis A. Rich A. Maas S. (2004). Widespread A-to-I RNA editing of Alu-containing mRNAs in the human transcriptome. *PLoS Biol.* 2:e391. 10.1371/journal.pbio.0020391 15534692 PMC526178

[B3] Baños-Lara M. D. R. Ghosh A. Guerrero-Plata A. (2013). Critical role of MDA5 in the interferon response induced by human metapneumovirus infection in dendritic cells and in vivo. *J. Virol.* 87 1242–1251. 10.1128/JVI.01213-12 23152520 PMC3554051

[B4] Barak M. Levanon E. Y. Eisenberg E. Paz N. Rechavi G. Church G. M. (2009). Evidence for large diversity in the human transcriptome created by Alu RNA editing. *Nucleic Acids Res.* 37 6905–6915. 10.1093/nar/gkp729 19740767 PMC2777429

[B5] Bazak L. Haviv A. Barak M. Jacob-Hirsch J. Deng P. Zhang R. (2014). A-to-I RNA editing occurs at over a hundred million genomic sites, located in a majority of human genes. *Genome Res.* 24 365–376. 10.1101/gr.164749.113 24347612 PMC3941102

[B6] Blanco-Melo D. Nilsson-Payant B. E. Liu W.-C. Møller R. Panis M. Sachs D. (2020). SARS-CoV-2 launches a unique transcriptional signature from in vitro, ex vivo, and in vivo systems. *bioRxiv* [Preprint]. 10.1101/2020.03.24.004655

[B7] Blow M. Futreal P. A. Wooster R. Stratton M. R. (2004). A survey of RNA editing in human brain. *Genome Res.* 14 2379–2387. 10.1101/gr.2951204 15545495 PMC534661

[B8] Cogné M. (2013). Activation-induced deaminase in B lymphocyte maturation and beyond. *Biomed. J.* 36 259–268. 10.4103/2319-4170.113191 24385067

[B9] Eisenberg E. Levanon E. Y. (2018). A-to-I RNA editing - immune protector and transcriptome diversifier. *Nat. Rev. Genet.* 19 473–490. 10.1038/s41576-018-0006-1 29692414

[B10] Free Software Foundation (2019). *GNU Operating System. Free Software Foundation, I.* Available online at: https://www.gnu.org/software/sed/ (accessed August 20, 2019).

[B11] Galeano F. Tomaselli S. Locatelli F. Gallo A. (2012). A-to-I RNA editing: the “ADAR” side of human cancer. *Semin. Cell. Dev. Biol.* 23 244–250. 10.1016/j.semcdb.2011.09.003 21930228

[B12] Gallo A. Locatelli F. (2012). ADARs: allies or enemies? The importance of A-to-I RNA editing in human disease: from cancer to HIV-1. *Biol. Rev. Camb. Philos. Soc.* 87 95–110. 10.1111/j.1469-185X.2011.00186.x 21682836

[B13] Ganem N. S. Ben-Asher N. Lamm A. T. (2017). In cancer, A-to-I RNA editing can be the driver, the passenger, or the mechanic. *Drug Resistance Updates* 32 16–22. 10.1016/j.drup.2017.09.001 29145975

[B14] Ganem N. S. Lamm A. T. (2017). A-to-I RNA editing - thinking beyond the single nucleotide. *RNA Biol.* 14 1690–1694. 10.1080/15476286.2017.1364830 28820319 PMC5731795

[B15] Goldstein B. Agranat-Tamir L. Light D. Ben-Naim Zgayer O. Fishman A. Lamm A. T. (2017). A-to-I RNA editing promotes developmental stage-specific gene and lncRNA expression. *Genome Res.* 27 462–470. 28031250 10.1101/gr.211169.116 PMC5340973

[B16] Kim D. D. Kim T. T. Walsh T. Kobayashi Y. Matise T. C. Buyske S. (2004). Widespread RNA editing of embedded alu elements in the human transcriptome. *Genome Res.* 14 1719–1725. 10.1101/gr.2855504 15342557 PMC515317

[B17] Kircheis R. Haasbach E. Lueftenegger D. Heyken W. T. Ocker M. Planz O. (2020). NF-kappaB pathway as a potential target for treatment of critical stage COVID-19 patients. *Front. Immunol.* 11:598444. 10.3389/fimmu.2020.598444 33362782 PMC7759159

[B18] Kleinberger Y. Eisenberg E. (2010). Large-scale analysis of structural, sequence and thermodynamic characteristics of A-to-I RNA editing sites in human Alu repeats. *BMC Genomics* 11:453. 10.1186/1471-2164-11-453 20667096 PMC3091650

[B19] Kleinman C. L. Majewski J. (2012). Comment on “widespread RNA and DNA sequence differences in the human transcriptome”. *Science* 335 1302–1302. 10.1126/science.1209658 22422962

[B20] Kung C. P. Maggi L. B. Jr. Weber J. D. (2018). The role of RNA editing in cancer development and metabolic disorders. *Front. Endocrinol. (Lausanne)* 9:762. 10.3389/fendo.2018.00762 30619092 PMC6305585

[B21] Langmead B. Trapnell C. Pop M. Salzberg S. L. (2009). Ultrafast and memory-efficient alignment of short DNA sequences to the human genome. *Genome Biol.* 10:R25. 10.1186/gb-2009-10-3-r25 19261174 PMC2690996

[B22] Lee S. Y. Joung J. G. Park C. H. Park J. H. Kim J. H. (2015). RCARE: RNA Sequence Comparison and Annotation for RNA Editing. *BMC Med. Genomics* 8(Suppl. 2):S8. 10.1186/1755-8794-8-S2-S8 26043858 PMC4460956

[B23] Levanon E. Y. Eisenberg E. Yelin R. Nemzer S. Hallegger M. Shemesh R. (2004). Systematic identification of abundant A-to-I editing sites in the human transcriptome. *Nat. Biotechnol.* 22 1001–1005. 10.1038/nbt996 15258596

[B24] Levanon K. Eisenberg E. Rechavi G. Levanon E. Y. (2005). Letter from the editor: Adenosine-to-inosine RNA editing in Alu repeats in the human genome. *EMBO Rep.* 6 831–835. 10.1038/sj.embor.7400507 16138094 PMC1369171

[B25] Li H. Handsaker B. Wysoker A. Fennell T. Ruan J. Homer N. (2009). The Sequence Alignment/Map format and SAMtools. *Bioinformatics (Oxford, England)* 25 2078–2079. 10.1093/bioinformatics/btp352 19505943 PMC2723002

[B26] Li M. Wang I. X. Li Y. Bruzel A. Richards A. L. Toung J. M. (2011). Widespread RNA and DNA sequence differences in the human transcriptome. *Science* 333 53–58. 10.1126/science.1207018 21596952 PMC3204392

[B27] Liddicoat B. J. Piskol R. Chalk A. M. Ramaswami G. Higuchi M. Hartner J. C. (2015). RNA editing by ADAR1 prevents MDA5 sensing of endogenous dsRNA as nonself. *Science* 349 1115–1120. 10.1126/science.aac7049 26275108 PMC5444807

[B28] Love M. I. Huber W. Anders S. (2014). Moderated estimation of fold change and dispersion for RNA-seq data with DESeq2. *Genome Biol.* 15:550. 10.1186/s13059-014-0550-8 25516281 PMC4302049

[B29] Maas S. Kawahara Y. Tamburro K. M. Nishikura K. (2006). A-to-I RNA editing and human disease. *RNA Biol.* 3 1–9. 10.4161/rna.3.1.2495 17114938 PMC2947206

[B30] Mannion N. M. Greenwood S. M. Young R. Cox S. Brindle J. Read D. (2014). The RNA-editing enzyme ADAR1 controls innate immune responses to RNA. *Cell Rep.* 9 1482–1494. 10.1016/j.celrep.2014.10.041 25456137 PMC4542304

[B31] Moore B. J. B. June C. H. (2020). Cytokine release syndrome in severe COVID-19. *Science* 368 473–474. 10.1126/science.abb8925 32303591

[B32] Osenberg S. Paz Yaacov N. Safran M. Moshkovitz S. Shtrichman R. Sherf O. (2010). Alu sequences in undifferentiated human embryonic stem cells display high levels of A-to-I RNA editing. *PLoS One* 5:e11173. 10.1371/journal.pone.0011173 20574523 PMC2888580

[B33] Paz-Yaacov N. Levanon E. Y. Nevo E. Kinar Y. Harmelin A. Jacob-Hirsch J. (2010). Adenosine-to-inosine RNA editing shapes transcriptome diversity in primates. *Proc. Natl. Acad. Sci. U.S.A.* 107 12174–12179. 10.1073/pnas.1006183107 20566853 PMC2901480

[B34] Picardi E. Manzari C. Mastropasqua F. Aiello I. D’Erchia A. M. Pesole G. (2015). Profiling RNA editing in human tissues: towards the inosinome Atlas. *Sci. Rep.* 5:14941. 10.1038/srep14941 26449202 PMC4598827

[B35] Picardi E. Pesole G. (2013). REDItools: high-throughput RNA editing detection made easy. *Bioinformatics* 29 1813–1814. 10.1093/bioinformatics/btt287 23742983

[B36] Piechotta M. Wyler E. Ohler U. Landthaler M. Dieterich C. (2017). JACUSA: site-specific identification of RNA editing events from replicate sequencing data. *BMC Bioinform.* 18:7. 10.1186/s12859-016-1432-8 28049429 PMC5210316

[B37] Pinto Y. Levanon E. Y. (2019). Computational approaches for detection and quantification of A-to-I RNA-editing. *Methods (San Diego, Calif.)* 156 25–31. 10.1016/j.ymeth.2018.11.011 30465820

[B38] Porath H. T. Carmi S. Levanon E. Y. (2014). A genome-wide map of hyper-edited RNA reveals numerous new sites. *Nat. Commun.* 5:4726. 10.1038/ncomms5726 25158696 PMC4365171

[B39] Quin J. Sedmik J. Vukic D. Khan A. Keegan L. P. O’Connell M. A. (2021). ADAR RNA modifications, the epitranscriptome and innate immunity. *Trends Biochem. Sci.* 10.1016/j.tibs.2021.02.002 [Epub ahead of print]. 33736931

[B40] Ramaswami G. Li J. B. (2014). RADAR: a rigorously annotated database of A-to-I RNA editing. *Nucleic Acids Res.* 42 109–113. 10.1093/nar/gkt996 24163250 PMC3965033

[B41] Ramaswami G. Zhang R. Piskol R. Keegan L. P. Deng P. O’Connell M. A. (2013). Identifying RNA editing sites using RNA sequencing data alone. *Nat. Methods* 10 128–132. 10.1038/nmeth.2330 23291724 PMC3676881

[B42] Rosenberg B. R. Hamilton C. E. Mwangi M. M. Dewell S. Papavasiliou F. N. (2011). Transcriptome-wide sequencing reveals numerous APOBEC1 mRNA-editing targets in transcript 3’ UTRs. *Nat. Struct. Mol. Biol.* 18 230–236. 10.1038/nsmb.1975 21258325 PMC3075553

[B43] Schaefer M. Kapoor U. Jantsch M. F. (2017). Understanding RNA modifications: the promises and technological bottlenecks of the ‘epitranscriptome’. *Open Biol.* 7:170077. 10.1098/rsob.170077 28566301 PMC5451548

[B44] Schneider W. M. Chevillotte M. D. Rice C. M. (2014). Interferon-stimulated genes: a complex web of host defenses. *Annu. Rev. Immunol.* 32 513–545. 10.1146/annurev-immunol-032713-120231 24555472 PMC4313732

[B45] Treangen T. J. Salzberg S. L. (2011). Repetitive DNA and next-generation sequencing: computational challenges and solutions. *Nat. Rev. Genet.* 13 36–46. 10.1038/nrg3117 22124482 PMC3324860

[B46] Vitali P. Scadden A. D. J. (2010). Double-stranded RNAs containing multiple IU pairs are sufficient to suppress interferon induction and apoptosis. *Nat. Struct. Mol. Biol.* 17 1043–1050. 10.1038/nsmb.1864 20694008 PMC2935675

[B47] Wang Z. Lian J. Li Q. Zhang P. Zhou Y. Zhan X. (2016). RES-Scanner: a software package for genome-wide identification of RNA-editing sites. *GigaScience* 5:37. 10.1186/s13742-016-0143-4 27538485 PMC4989487

[B48] Wu D. Lamm A. T. Fire A. Z. (2011). Competition between ADAR and RNAi pathways for an extensive class of RNA targets. *Nat. Struct. Mol. Biol.* 18 1094–1101. 10.1038/nsmb.2129 21909095 PMC3190075

[B49] Wu J. Xie F. Qian K. Gibson A. W. Edberg J. C. Kimberly R. P. (2011). FAS mRNA editing in human systemic lupus erythematosus. *Hum. Mutat.* 32 1268–1277. 10.1002/humu.21565 21793106 PMC3196739

[B50] Yin X. Riva L. Pu Y. Martin-Sancho L. Kanamune J. Yamamoto Y. (2021). MDA5 governs the innate immune response to SARS-CoV-2 in lung epithelial cells. *Cell Reports* 34:108628. 10.1016/j.celrep.2020.108628 33440148 PMC7832566

[B51] Zhang Q. Xiao X. (2015). Genome sequence-independent identification of RNA editing sites. *Nat. Methods* 12 347–350. 10.1038/nmeth.3314 25730491 PMC4382388

[B52] Zhu S. Xiang J.-F. Chen T. Chen L.-L. Yang L. (2013). Prediction of constitutive A-to-I editing sites from human transcriptomes in the absence of genomic sequences. *BMC Genomics* 14:206. 10.1186/1471-2164-14-206 23537002 PMC3637798

[B53] Zuberi K. Franz M. Rodriguez H. Montojo J. Lopes C. T. Bader G. D. (2013). GeneMANIA prediction server 2013 update. *Nucleic Acids Res.* 41 W115–W122. 10.1038/nmeth.3314 23794635 PMC3692113

