# Supplementary material for: RESIC: A Tool for Comprehensive Adenosine to Inosine RNA Editing Site Identification and Classification
Source: Front Genet. 2021 Jul 23;12:686851. doi: 10.3389/fgene.2021.686851 (PMC8343188; doi:10.3389/fgene.2021.686851)
Supplement: Supplementary file 1 [file Data_Sheet_1.pdf]

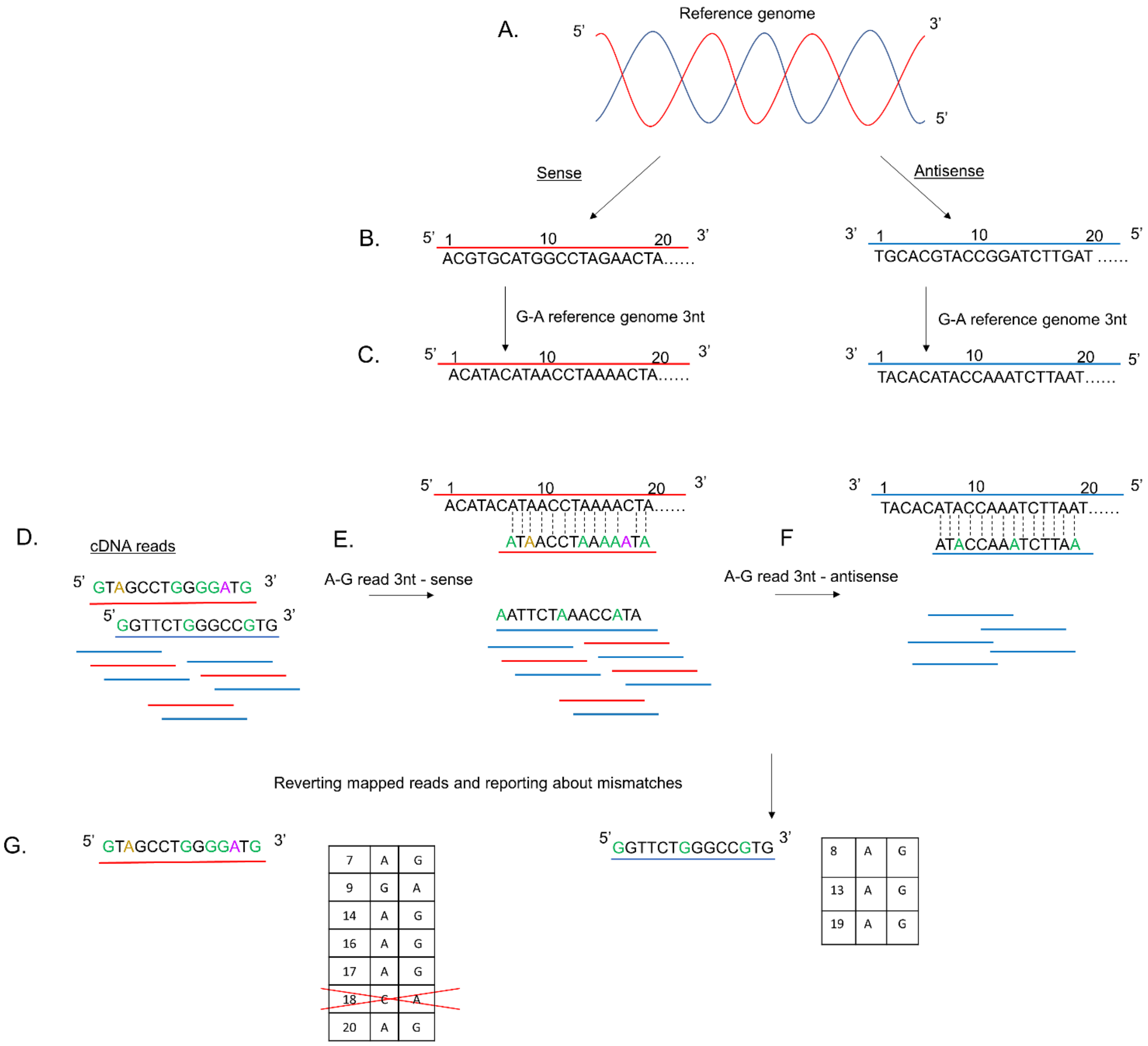

Figure S1: Detailed illustration of 3nt genome alignment scheme. (A) A reference genome. **(B)** The DNA sequence of the reference genome split by strand. Red, sense strand, presented in 5'-to-3' direction; Blue, antisense strand, presented in 3'-to-5' direction. The nucleotide sequences complement each other. **(C)** G-A 3nt replacement for the reference genome: Gs are converted into As **(D)** cDNA reads from RNA-sequencing, all obtained in 5'-to-3' direction. Green nucleotides indicate for A-to-G editing sites (G instead of A in the reference genome). Brown nucleotides present G-to-A changes compared to the reference genome. Purple nucleotides present other mismatches than A-to-G or G-to-A. Red, sense strand; Blue, antisense strand. **(E)** Alignment following 3nt graph scheme - Part 1. read 3nt sense: Gs are converted into As in the cDNA reads. Hyper edited sense reads are successfully being mapped to the reference genome. **(F)** Alignment following 3nt graph scheme- Part 2, 3nt antisense: unmapped reads from part 1 are being reverted (not shown) and reversed to fit the 3'-to-5' direction of the antisense reference strand. Gs are converted into As in the cDNA reads. Hyper edited antisense reads are successfully being mapped to the reference genome. **(G)** Reverting mapped reads back from Gs to As and defining editing sites. Sites other than A-to-G or G-to-A are excluded.

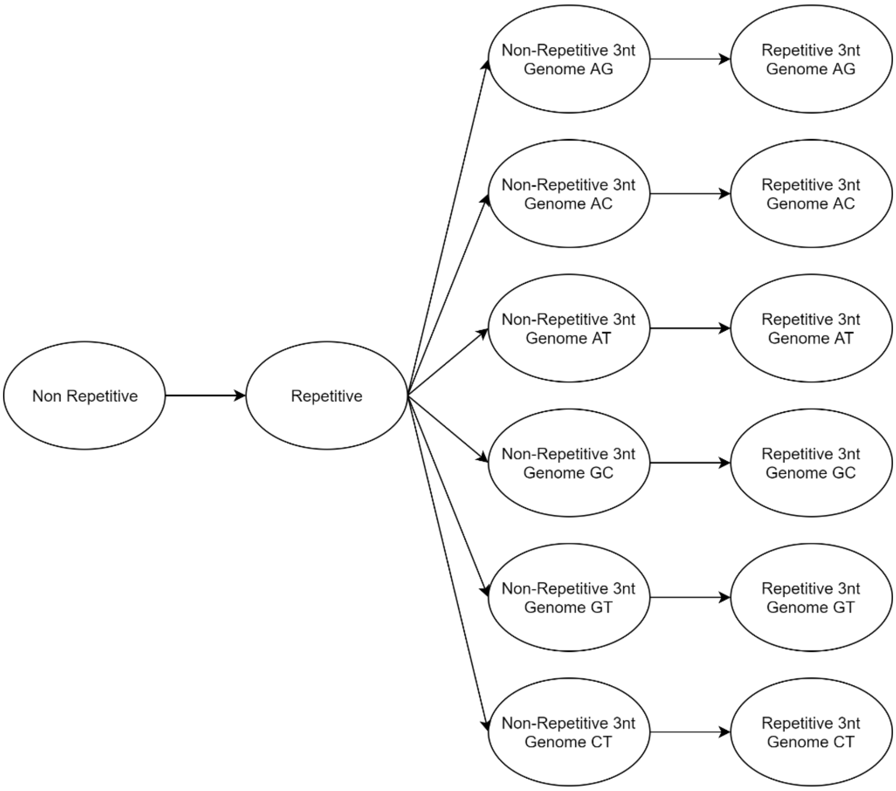

Figure S2: **RESIC graph alignment.** Non-repetitive alignment is followed by repetitive alignment. This is followed by 3nt Genome scheme with non-repetitive alignment parameters and 3nt Genome scheme with repetitive alignment parameters for each pair of nucleotides.

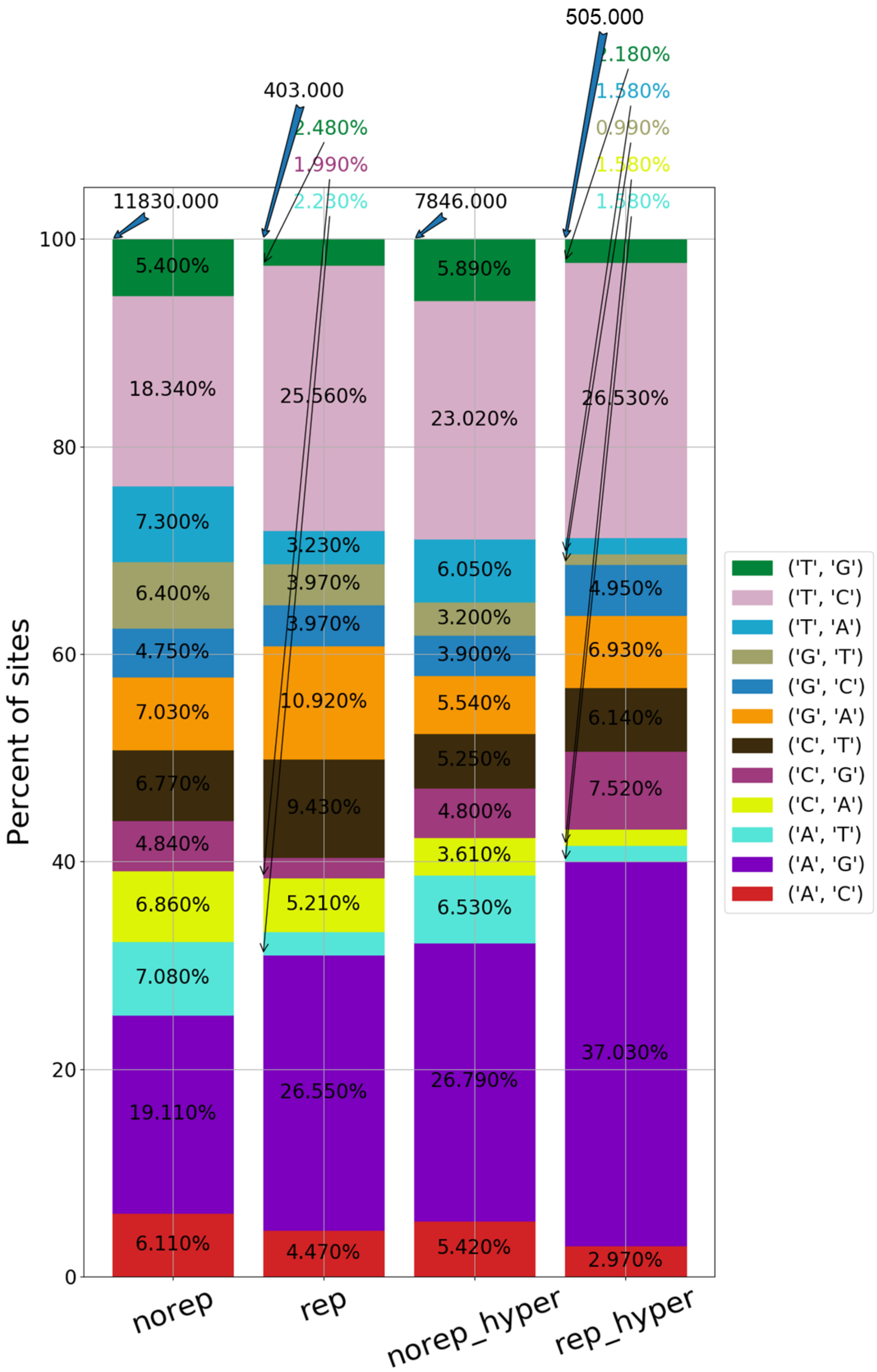

Figure S3: RESIC editing percent distribution plot, obtained for the adipose tissue. Blue arrow at the top of each bar shows the total number of sites being identified for the class. The percentages on the bars present the total number of editing type out of all identified site in the class.

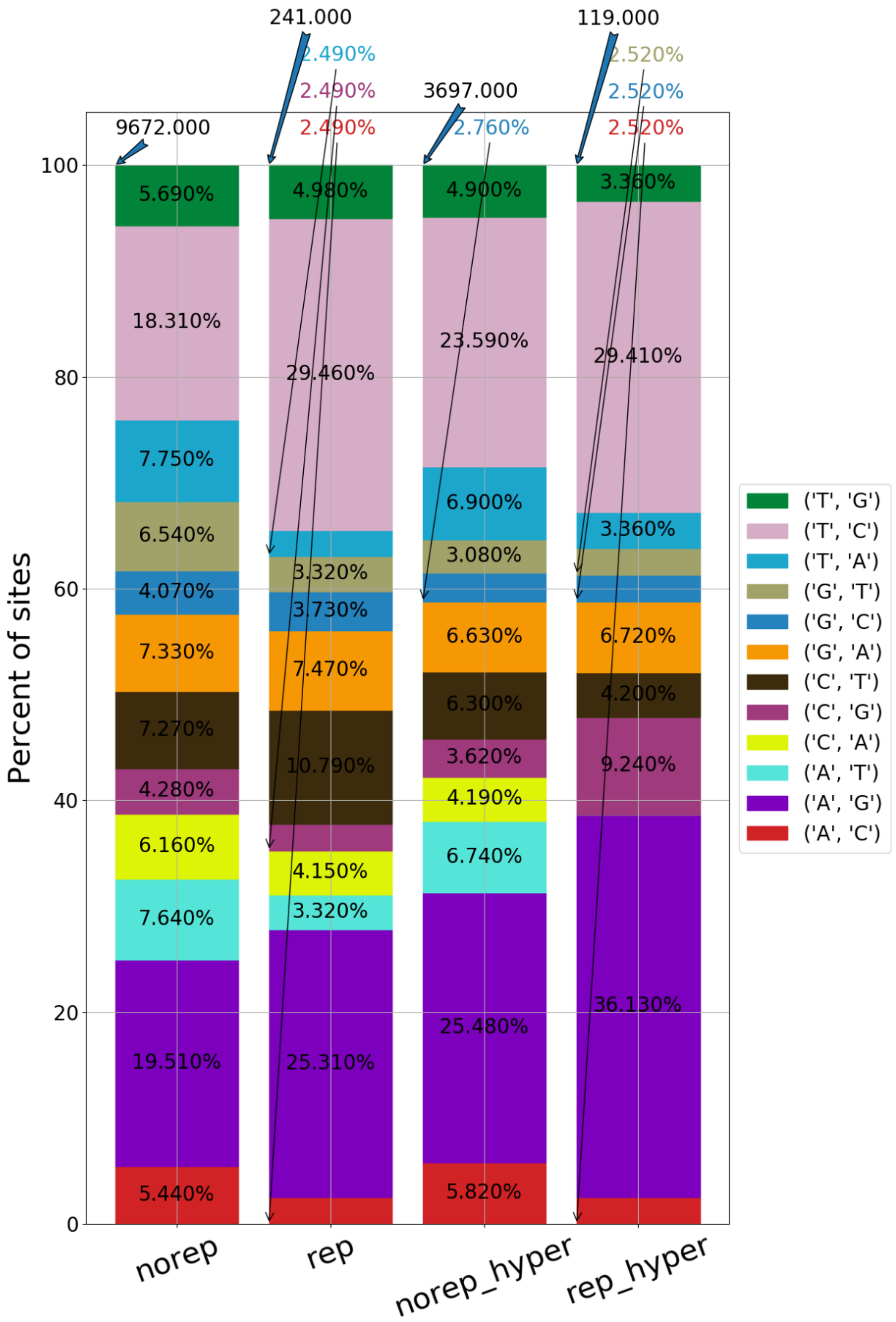

FigureS4 :RESIC editing percent distribution plot, obtained for the brain tissue. Blue arrow at the top of each bar shows the total number of sites being identified for the class. The percentages on the bars present the total number of editing type out of all identified site in the class.

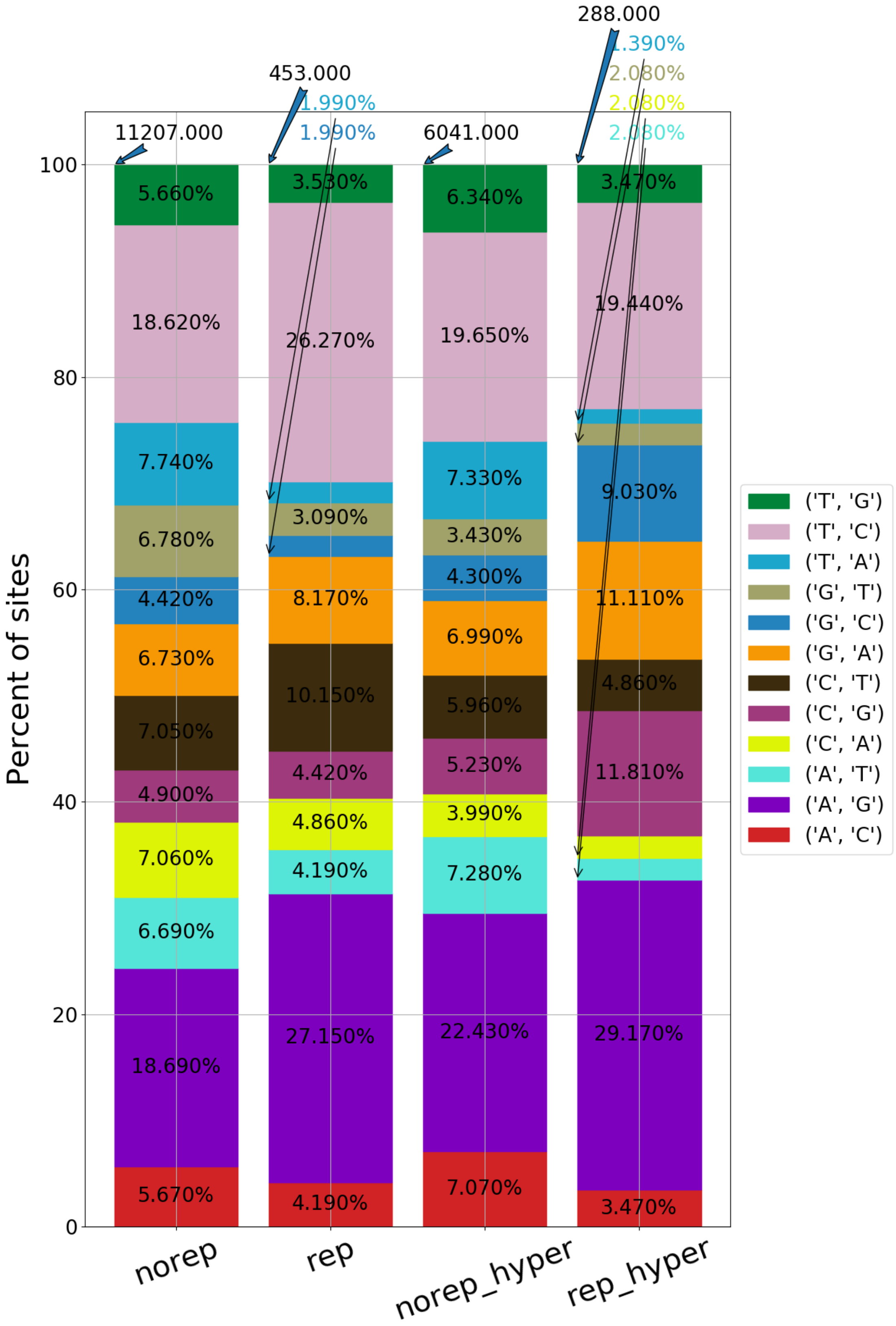

FigureS5 :RESIC editing percent distribution plot, obtained for the breast tissue. Blue arrow at the top of each bar shows the total number of sites being identified for the class. The percentages on the bars present the total number of editing type out of all identified site in the class.

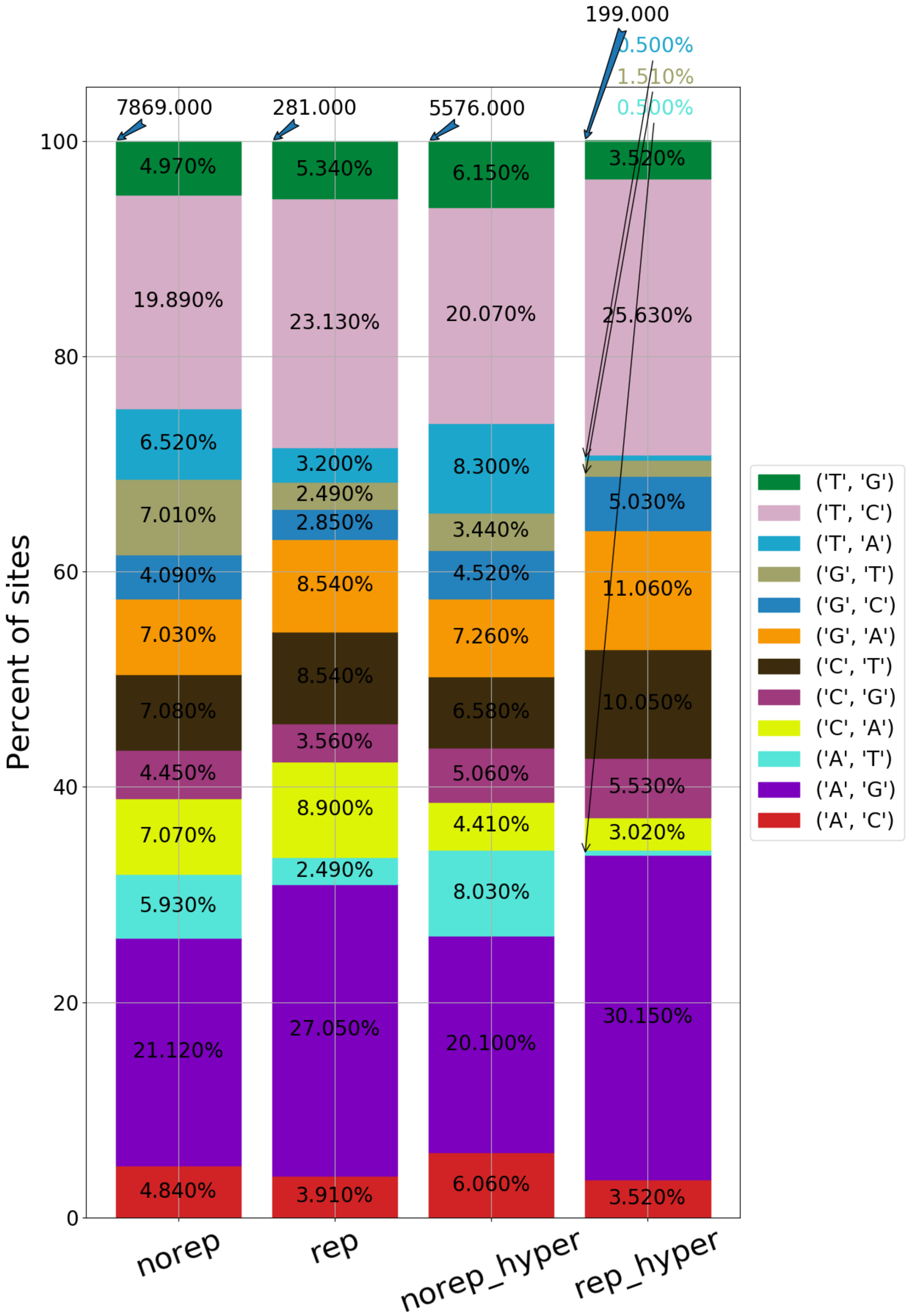

FigureS6 :RESIC editing percent distribution plot, obtained for the colon tissue. Blue arrow at the top of each bar shows the total number of sites being identified for the class. The percentages on the bars present the total number of editing type out of all identified site in the class.

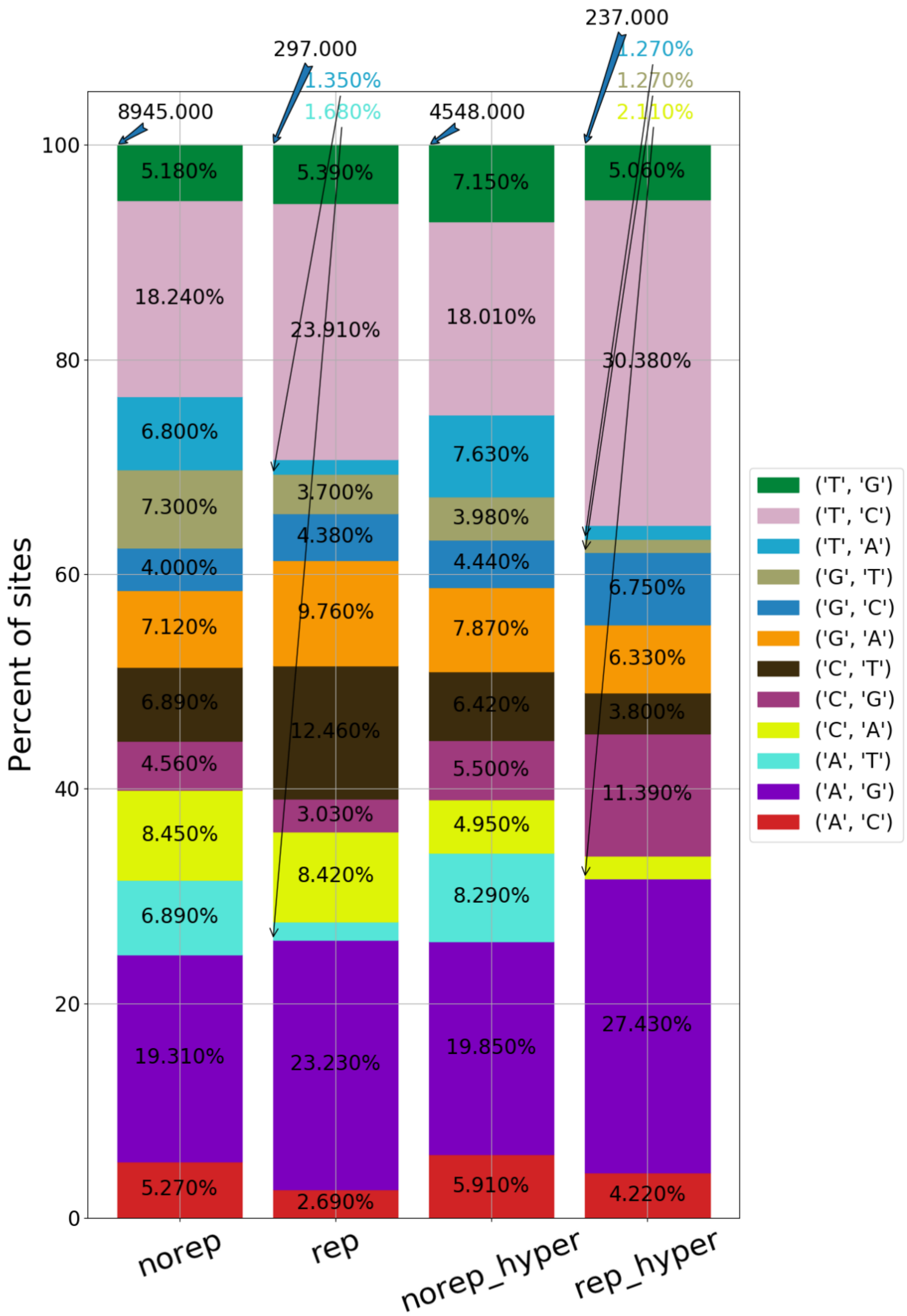

FigureS7 :RESIC editing percent distribution plot, obtained for the kidney tissue. Blue arrow at the top of each bar shows the total number of sites being identified for the class. The percentages on the bars present the total number of editing type out of all identified site in the class.

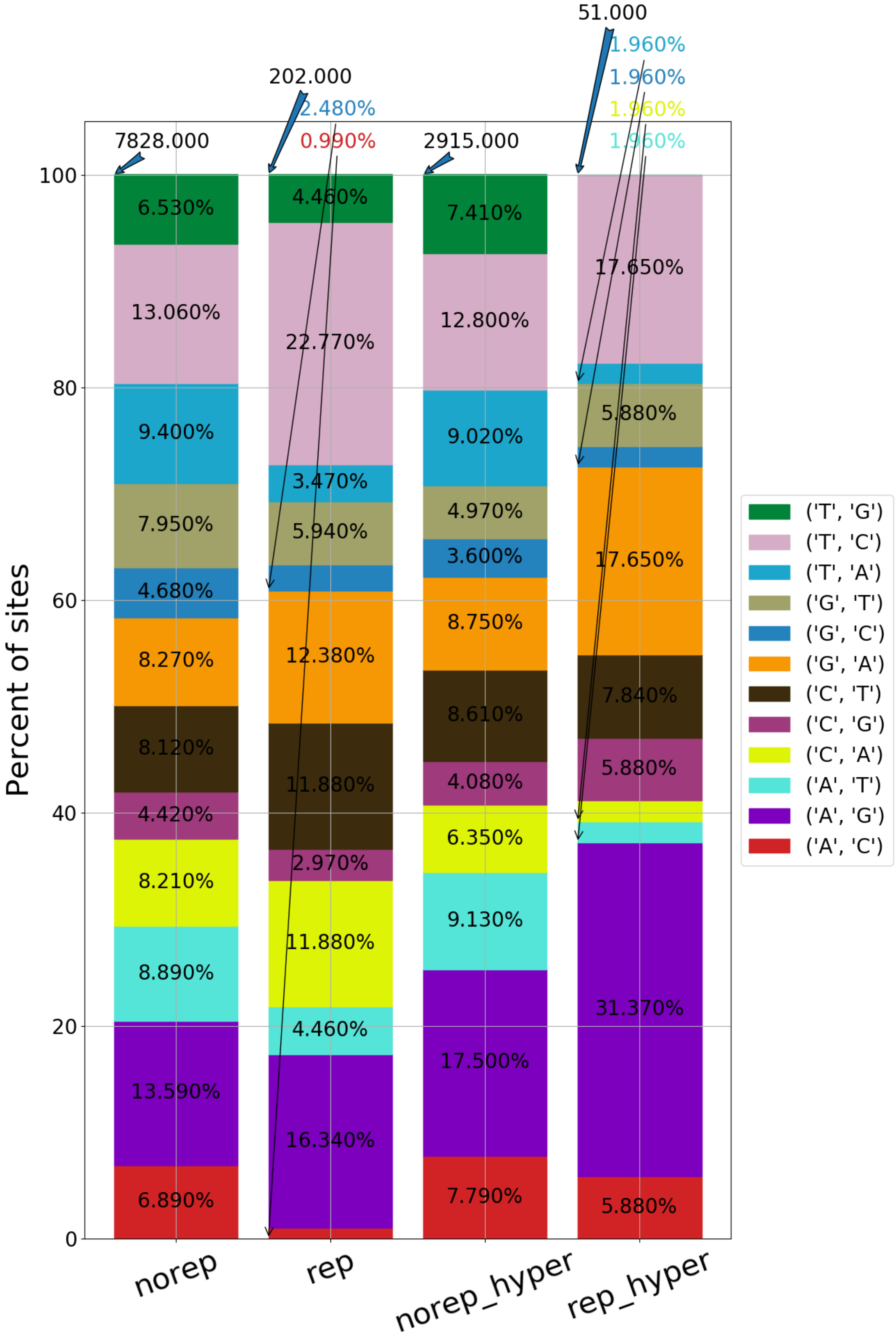

FigureS8 :RESIC editing percent distribution plot, obtained for the heart tissue. Blue arrow at the top of each bar shows the total number of sites being identified for the class. The percentages on the bars present the total number of editing type out of all identified site in the class.

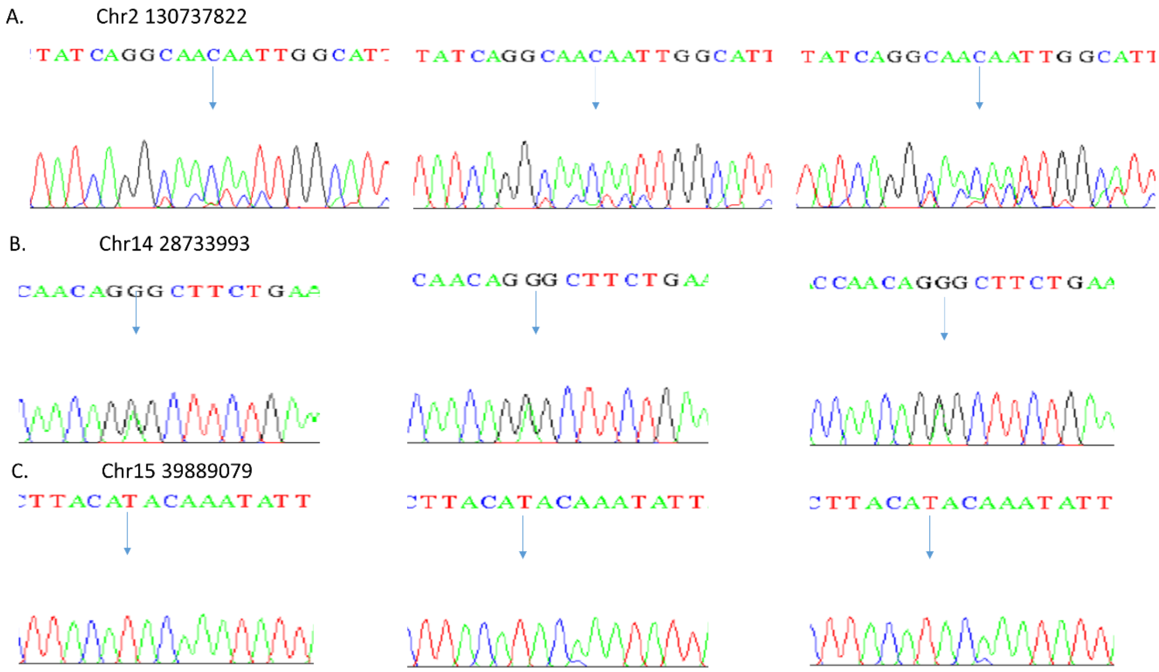

FigureS9: Validating RNA editing in novel sites found by RESIC using Sanger sequencing. Sanger sequencing of RNA from three sections of brain glioblastoma tissue. The predicted editing site is marked in a blue arrow. **A.** Validation for editing event (a change from T to C) in chromosome 2, position 130737822, *RAB6C* gene. **B.** Validation for editing event (a change from A to G) in chromosome 14, position 28733993 **C.** No editing was recorded in the predicted editing site in chromosome 15, position 39889079, *THBS1* gene in sanger sequencing.

Primers used for RNA editing validation using Sanger sequencing

Primer sequence	Target editing site location	Primer direction
AATCCGCTGAGGAAATTC	chromosome 2 130737822	F
CTACAGCTGCAGCAGAATC		R
TTTCTATTTATAATGGTGACATGG	chromosome 4 28733993	F
ACTTGACCAACCCATATCC		R
TGCAAAGAAAGCCATGAG	chromosome 15 39889079	F
TCTGCAGAGTTTTTATGCTATGTAC		R

Supplementary table legend

Supplementary Table S1: the list of editing sites across different classes identified using samples from the Illumina Human Body Map project.

Supplementary Table S2: characterization of editing landscape with respect to site locations and gene annotation

Supplementary Table S3: unique genes under the SARS-CoV-2 nonrepetitive hyper-editing class and GO enrichment analysis.

Supplementary Table S4: unique genes under the SARS-CoV-2 nonrepetitive class and GO enrichment analysis.

Supplementary Table S5: unique genes under the mock nonrepetitive hyper-editing class and GO enrichment analysis.

Supplementary Table S6: unique genes under the mock nonrepetitive class and GO enrichment analysis.

Supplementary Table S7: differential expression analysis for SARS-CoV-2 infected samples versus mock, in the Calu3 cell line.

Supplementary Table S8: differential expression results of ADAR1 and ADAR2 genes, for SARS-CoV-2 infected samples versus mock, in A549 and NHBE cell lines.
